# Supplementary material for: Assessment of hepatitis B vaccination status and hepatitis B surface antibody titres among health care workers in selected public health hospitals in Kenya
Source: PLOS Glob Public Health. 2023 Apr 12;3(4):e0001741. doi: 10.1371/journal.pgph.0001741 (PMC10096287; doi:10.1371/journal.pgph.0001741)
Supplement: S1 Appendix — (PDF) [file pgph.0001741.s001.pdf]

Appendix 2: Immunization history questionnaire for health care workers

Hospital Name: _________________________ Study Participant ID: ___________________

For classification purpose only:

1. Gender □ Male □ Female

2. Age

   □ 18 to 25 years□ 26 to 35 years□ 36 to 45 years□ 46 to 55 years□ Over 56 years

3. How long have you worked in the present hospital?

   □ 1 to 2 years □ 3 to 5 years □ 6 to 10 years □ Over 11 years

4. What is your role in the hospital? (e.g. nurse, lab technician, doctor.)

   ____

KENYATTA NATIONAL HOSPITAL
APPROVED
28 JUN 2016
KNH/UoN-ERC
P.O.Box 20723-00202 NRB.

Immunization and HBV diagnosis history:

5. What immunizations have you received? (Tick the appropriate box)

a) Measles Mumps and Rubella (MMR)	□ Yes	□ No	□ I'm not sure
b) Hepatitis A	□ Yes	□ No	□ I'm not sure
c) Hepatitis B	□ Yes	□ No	□ I'm not sure
d) Tetanus, Diphtheria, Pertussis (last 2 years)	□ Yes	□ No	□ I'm not sure
e) Influenza (Flu)	□ Yes	□ No	□ I'm not sure
f) Varicella (Chicken pox)	□ Yes	□ No	□ I'm not sure
g) Yellow fever	□ Yes	□ No	□ I'm not sure
h) Rabies	□ Yes	□ No	□ I'm not sure

i) Others (please specify) ____

6. Do you have records for all the immunizations above that you have received?

□ Yes □ No □ Some (specify) ______________________

7. Are you immunized against HBV? If yes, when were you immunized?

□ No □ Yes (Specify) ______________________

4. How many doses of the vaccine did you receive?

____

5. Were you tested for hepatitis B immunity status post vaccination?

□ Yes □ No

6. Have you ever been diagnosed for HBV infection?

□ Yes □ No

If yes what was the results? □ Negative □ Positive

KENYATTA NATIONAL HOSPITAL
APPROVED
28 JUN 2016
KNH/UoN-ERC
P.O. Box 20723-00202 NRB.

Knowledge and attitude

7. Should health care workers need extra vaccinations for protection and safety?

□ I agree □ I disagree □ Not sure

If you agree do you think the vaccines are readily available? □ Yes □ No □ Not sure

8. Where vaccines are available should there be regulations to enforce immunization requirements to all health care workers?

□ I agree □ I disagree

9. Is it important for all health care workers to receive HBV immunization?

□ Yes □ No

10. In your role at the hospital, what are the possible risks of HBV infection?

____

11. Would you voluntarily receive a HBV vaccine if provided for?

□ Yes □ No

12. Why would you not wish to receive the HBV vaccine? (Tick all that apply)

a) Fear of needles	□ I agree	□ I disagree
b) Concerns about safety of vaccine	□ I agree	□ I disagree
c) I am not in high risk group	□ I agree	□ I disagree
d) I never get sick	□ I agree	□ I disagree
e) If pregnant at time of vaccination	□ I agree	□ I disagree
f) Religious opposition to vaccination	□ I agree	□ I disagree
g) I got sick the last time I was vaccinated	□ I agree	□ I disagree

h) Other reasons (Please specify) ______________________________

Thank you for your time and help with this important study!

KENYATTA NATIONAL HOSPITAL
APPROVED
28 JUN 2016
KNH/UON-ERC
P.O. Box 20723-00202 NRB.
