# Supplementary material for: Assessment of hepatitis B vaccination status and hepatitis B surface antibody titres among health care workers in selected public health hospitals in Kenya
Source: PLOS Glob Public Health. 2023 Apr 12;3(4):e0001741. doi: 10.1371/journal.pgph.0001741 (PMC10096287; doi:10.1371/journal.pgph.0001741)
Supplement: Attachment — Submitted filename: RESPONSES_To Reviewers comments.docx [file pgph.0001741.s003.docx]

Irene Ann Mwangi,

Institute of Tropical Medicine and Infectious Diseases,

JOMO Kenyatta University of Agriculture and Technology (JKUAT),

P.O BOX 54840-00200,

Nairobi.

Tel: +254 725398697

Email: [wiann97@yahoo.com](mailto:wiann97@yahoo.com)

22^nd^ December 2022

**To**
**The Staff Editor,**

**PLOS Global Public Health Scientific Journal**
[globalpubhealth@plos.org](mailto:globalpubhealth@plos.org) / [aschaefer@plos.org](mailto:aschaefer@plos.org)

Dear Editor,

We thank you and all the reviewers for the valuable queries and suggestions; we are delighted that PLOS Global Public Health will consider publication of our Manuscript ID PGPH-D-22-01550 pending satisfactory revisions as suggested by the reviewers.

We have given careful consideration to all the reviewers’ comments and have done our best to address them all. All our changes are highlighted in track changes within the revised Main document.

The following is a point by point explanation of how we have addressed the reviewers’ questions and comments.

**Reviewer: 1**

1. Comment

Avoid abbreviations in the abstract

Response

Thank you for the suggestion. Due to the limited number of words required for the abstract section by the journal (300 words), we are unable to completely avoid abbreviations. We have however spelt out the full term for every abbreviation used.

2. Comment

I recommend more description about the statistical analysis in the abstract sections

Response

Thank you for the suggestion. However, the number of words acceptable for the abstract section limits us from adding more information into this section. Kindly accept statistical details as provided under Methods section, Statistical analysis lines 127-135

3. Comments 3, 6, 7, 8

I think, it is not the matter of vaccination for health workers as the survey is done on health professionals rather than the completeness of the vaccination (fully vaccinations) and immunization status. So better to focus on the immunization status

I recommend focusing immunization status rather than vaccination status

Line 195: The same is true in line 195 as I have mentioned before

In table2: it is meaningless to compare the immunization status between vaccinated with unvaccinated ones. The comparison should be between fully vaccinated and not fully vaccinated

General comment through email: Authors should address all the points to consider acceptance for publication. I recommend to focus the immunization status than vaccination status. Theoretical, there is no immunization without the exposure for an antigen

Response

Thank you for this guidance. It is indeed true that immunization cannot occur without exposure to an antigen. In this paper, we are cognisant of the fact that exposure to an antigen can either be through natural infection or through vaccines which are either made of the whole structure or parts of the disease causing organism. The objective of the study was to assess vaccination status to inform on vaccine uptake rates as well as evaluate association between vaccine doses received and anti-HBs immune responses. Vaccine status was therefore an independent variable expected to influence seroprotection status (immunity) of the health care workers. Additionally, not all health care workers have access to hepatitis B vaccine in Kenya and it was therefore important to assess coverage. Seroprotection status was evaluated serologically through measurement of hepatitis B surface antibody (anti-HBs) titres followed by classification of responses based on Center for Disease control and Prevention (CDC) guidelines, where protection is defined as anti-HBs titres greater than 10 mIU/ml. All study participants were also tested for presence of antibodies to hepatitis B core antigen (anti-HBc) to evaluate natural exposure to the antigen (infection) and this informed why some unvaccinated individuals recruited into the study had positive anti-HBs responses.

To avoid ambiguity that would arise by using the term immunization status which in some instances is defined as to whether or not an individual has received certain vaccines, vaccination status and natural infection by the virus were both considered as independent variables that would elicit hepatitis B surface antibodies in an individual. We therefore request to retain “vaccination status” for this paper.

4. Comment

In line 20: “This study therefore aimed to assesses hepatitis B vaccination and sero-protection status among HCWs in three selected public hospitals in Kenya”. It should be written as “hepatitis B vaccination status”. This phrase should be uniform throughout the documents otherwise it gives different meaning and will be ambiguous for readers.

Response

Thank you. This has been corrected. Lines 20, 84, 197

5. Comment

In line 35: “Majority of the HCWs were vaccinated and seroprotected against hepatitis B virus”

The conjunction ‘and’ is not appropriate in this case because the level of vaccination is not equal to equal to the level of immunization to say ‘majority’ for both. So, amend it throughout the document

Response

Thank you for the guidance. This has been rephrased. Lines 36-37,261-262

**Reviewer: 2**

Comment:

Diligent study and HCW has to give great care while performing their duties because it is hazardous condition that should be treated within time and HCW are at great risk as they are exposing by the time to the patients.
Government has to take action for taking care of vaccination of HCW

Response:

Thank you for the comment. Indeed the government should ensure that all health care workers are vaccinated against Hepatitis B virus.

**Reviewer: 3**

Comments:

I am grateful for the opportunity to review the manuscript entitled “Assessment of Hepatitis B vaccination status and Hepatitis B surface antibody titres among health care workers in selected public health hospitals in Kenya.” The authors have assessed the hepatitis vaccination and seroprotection status of health care workers in the Kenyan population. This study is important because healthcare workers are more prone to hepatitis B infection than the general populace and there is a need to continue to sensitize health care workers and encourage full vaccination according to the recommended vaccination schedule. However, the following minor comments need to be addressed:

**Methods**
Comment: P.5, lines 103-104, kindly give the detail of how serum was separated from the whole blood samples collected from study participants. What centrifugation speed was used, for how long did you spin and what kind of bottles were the serum samples aliquoted into before storage at -80 ºC?
Response: Thank you for notifying us of this omission. We have added information on centrifugation and serum storage bottles under ‘data and samples collection’ P.5, lines 105-107. The section now reads as follows:

“Medical and socio-demographic data was collected using an interviewer-guided questionnaire after obtaining written informed consent. Health care workers who had received one or more hepatitis B vaccine doses were considered to be vaccinated; those who received the three recommended doses were considered to be fully vaccinated. Using Beckton Dickson serum separating tube (BD SST), 5mls of blood sample was collected from health care workers by a trained nurse. Samples from KNH and Mbagathi county hospitals were transported in a cool box to Kenya AIDs Vaccine Initiative–Institute of Clinical Research (KAVI–ICR), University of Nairobi for serum separation and storage while those from Naivasha sub-county hospital were centrifuged on site and serum transported to KAVI-ICR in dry ice. Centrifugation of all whole blood samples was done for 10 minutes at a speed of 3000 revolutions per minute, serum aliquoted into 2mls sterile Sarstedt screw cap microtubes and stored at -80ºC awaiting analysis.”

**Results**
Comment: P.10, lines 185-186, “Females were 2.8 times more likely to have anti-HBs compared to males.” This is an important finding which was not included in the discussion section. The authors need to explain why females were more likely to have anti-HBs compared to males in the study.

Response: Thank you for the suggestion. We have added information explaining why females were more likely to have anti-HBs compared to males under Discussion section P.13, lines 233-241.

“Our study revealed that females were more likely to be responders compared to males. The findings are in agreement with similar studies among health care workers in India and Rwanda where male non-response ranged from 13.7-16% while female non-response from 5.3-7.9% [9,10]. Trevisan et.al study on sex disparity in response to Hepatitis B vaccine also reported that females showed 20% higher vaccination-related antibody titres than males [19]. On the contrary, a different study by Basireddy et.al in India showed no association between sex and the rate seroconversion to anti-HBs [20]. It is hypothesized that smoking, alcohol and certain genetic factors are the probable contributors of poor responses in males [9,10,19]; we did not investigate this in our study.”

**Discussion**
Comment: P.13, line 226, please correct “The high percentages of non-response was concern as the study revealed” to “The high percentages of non-response was of concern as the study revealed”

Response: Thank you for the correction. The statement has been edited to read: “The high percentage of non-response was of concern as the study revealed.” P.13, line 229.

**Reference List**

We wish to inform you that we have added two references into the reference list as below. The new references have been cited in discussion section on P.13, lines 341-347.

19.	Trevisan A, Giuliani A, Scapellato ML, Anticoli S, Carsetti R, Zaffina S, et al. Sex disparity in response to hepatitis B vaccine related to the age of vaccination. Int J Environ Res Public Health. 2020;17(1).

20.	Basireddy P, Avileli S, Beldono N, Gundela S. Evaluation of immune response to hepatitis B vaccine in healthcare workers at a tertiary care hospital. Indian J Med Microbiol [Internet]. 2018;36(3):397–400. Available from: https://doi.org/10.4103/ijmm.IJMM_17_431
